# Cytokine networks and therapeutic advances in systemic lupus erythematosus

**DOI:** 10.3389/fimmu.2025.1680418

**Published:** 2025-11-07

**Authors:** Zeping Chen, Shupei Liu, Rui Xie, Pan Zhang, Yue Feng

**Affiliations:** 1 School of Acupuncture and Tuina, Chengdu University of Traditional Chinese Medicine, Chengdu, China; 2 Department of Tuina, Chengdu Pidu District Hospital of Traditional Chinese Medicine, Chengdu, China; 3 Department of Neurological Center, Traditional Chinese Medicine Hospital of Meishan, Meishan, China

**Keywords:** systemic lupus erythematosus, cytokine, Th17 cells, precision medicine, immunopathology

## Abstract

Systemic lupus erythematosus (SLE) is an autoimmune disease characterized by the loss of immune tolerance, leading to the production of autoantibodies and widespread organ damage. Th1, Th2, and Th17 cytokines play critical roles in driving inflammation and tissue injury in SLE. IL-17 and IL-23 have been identified as key mediators in disease progression, with ongoing clinical trials assessing the efficacy of their inhibitors. Additionally, cytokines like IL-10 and IL-22 exhibit dual roles, influencing both pathogenic and protective immune responses. While targeted therapies have shown clinical promise, challenges related to safety and long-term efficacy persist. Emerging targets such as MIF and IL-39 offer new insights into disease mechanisms. This review summarizes the immunoregulatory functions of these cytokines, focusing on their contributions to disease pathogenesis and potential therapeutic strategies, highlighting the importance of cytokine in SLE treatments.

## 1 Introduction

Systemic lupus erythematosus (SLE) is a persistent autoimmune condition that manifests with extensive organ involvement. Its pathological features are primarily defined by uncontrolled autoantibody synthesis, abnormal complement activation, and diffuse immune complex deposition across tissues (1, 2). The disease exhibits substantial heterogeneity, and its underlying mechanisms remain only partially understood. Current findings suggest that the interplay of genetic susceptibility, hormonal factors, environmental influences, and immune imbalances jointly fuels disease onset and progression (1, 3). Of particular importance, alterations in the functional states of CD4^+^ T helper (Th) cell subsets, along with disruption of their cytokine signaling networks, have been strongly linked to both disease pathogenesis and clinical expression (4).

On an immunological level, Th cells serve as key modulators of both innate and adaptive immunity through the secretion of lineage-defining cytokines. Studies have highlighted that proinflammatory mediators—including interleukin (IL)-12, IL-17, and IL-23—initiate feed-forward inflammatory circuits by acting on fibroblasts, epithelial cells, and various immune components. This interaction promotes the pathological release of chemokines, matrix metalloproteinases (MMPs), and other inflammatory molecules, thereby sustaining tissue injury and chronic inflammation seen in SLE (5). In this review, we focus on dissecting the immunoregulatory roles and clinical translational relevance of Th1-, Th2-, and Th17-associated cytokines under conditions of Th cell imbalance. Our goal is to elucidate the mechanistic underpinnings of cytokine-mediated immune dysregulation in SLE and to provide a foundation for more refined therapeutic strategies.

## 2 Th1-associated cytokines in SLE pathogenesis

### 2.1 Aberrant interferon signaling in SLE

**Figure 1:**
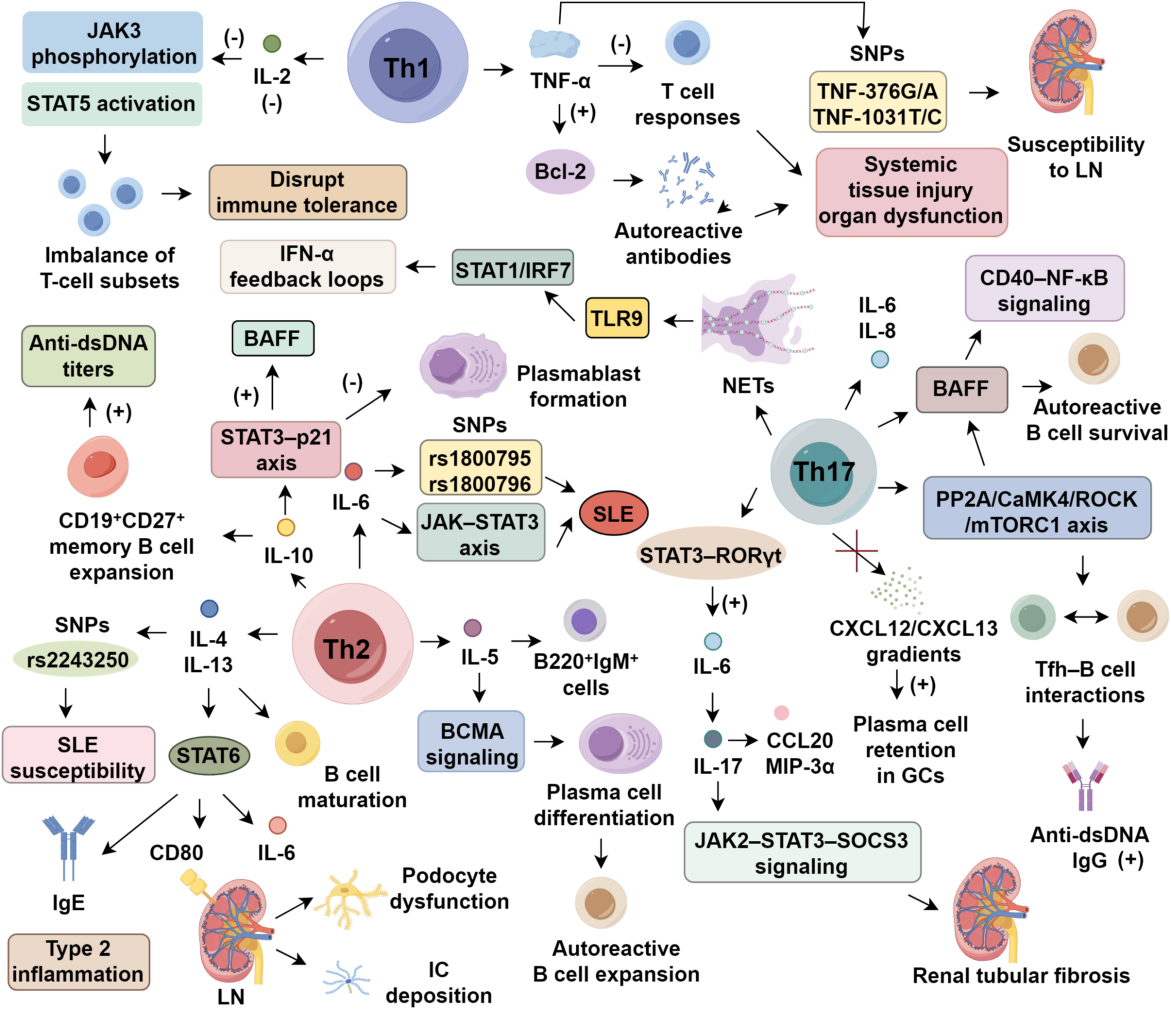
Cytokine regulation in systemic lupus erythematosus.

Dysregulated interferon-α (IFN-α) production via Toll-like receptor (TLR) signaling is a hallmark of SLE, with genetic polymorphisms in the IFN-α kinase cascade driving sustained type I interferon activation (6). Elevated serum IFN-α is linked to cutaneous lesions and lupus nephritis (LN) (7, 8), with levels paralleling disease activity indices like SLEDAI (9). Blocking IFN-α has shown clinical benefit: anifrolumab, an anti-IFN-α monoclonal antibody, achieved satisfied SRI-4 response in most patients during phase III trials, though long-term efficacy remains uncertain (10). In contrast, IFN-γ’s pathogenic relevance is less established. In murine lupus models, IFN-γ enhances anti-dsDNA production via FcγRIII-mediated complement activation, worsening renal pathology (11). Clinically, newly diagnosed SLE patients often exhibit elevated IFN-γ, correlating with anti-TPO antibody presence (12, 13). It is proposed that IFN-α drives early innate immune dysregulation, while IFN-γ contributes to adaptive immune activation during later stages (12, 14). However, the mechanisms—particularly involving Th1/Th17 interplay—underlying IFN-γ’s pathogenicity remain to be fully elucidated (**Figure 1**).

### 2.2 Bidirectional immunomodulation by IL-2 in SLE

IL-2 plays a dual role in SLE, where its deficiency disrupts immune tolerance while low-dose supplementation restores Treg homeostasis (15, 16). Impaired IL-2 signaling in SLE, characterized by diminished JAK3 phosphorylation and STAT5 activation, destabilizes T cell subset equilibrium (16). Therapeutic reconstitution with low-dose IL-2 selectively expands CD25^+^ regulatory T cells (Tregs) while limiting pathogenic helper and follicular helper T cells (Tfh), thereby reestablishing immune balance (16, 17). A randomized trial demonstrated that low-dose IL-2 significantly improved clinical outcomes within 6 months, with over 50% complete remission in LN and limited adverse events (18). To enhance efficacy and selectivity, IL-2/anti–IL-2 monoclonal antibody complexes have been developed, preferentially activating CD25^+^ Tregs while sparing effector T cells. In murine lupus models, these complexes boosted Treg proliferation and mitigated renal damage (19, 20). Next-generation IL-2 analogs—including muteins and pegylated variants such as bempegaldesleukin (NKTR-358)—demonstrate improved pharmacokinetics and sustained Treg expansion, with early clinical data suggesting favorable tolerability (21, 22). These strategies represent promising avenues for inducing durable immune tolerance in SLE.

### 2.3 TNF-α-driven inflammatory circuits

TNF-α functions as a multifunctional cytokine with paradoxical roles in SLE pathogenesis. Its deleterious effects are primarily mediated through the activation of macrophages by abnormal immune complexes, which induce TNF-α production and establish a proinflammatory milieu (23, 24). This, in turn, facilitates the generation of autoreactive antibodies by dampening T cell responses and enhancing the expression of anti-apoptotic regulators from the BCL-2 family, thereby contributing to systemic tissue injury and organ dysfunction (25). Clinical investigations underscore the aberrant regulation of TNF-α in SLE. Plasma concentrations of TNF-α were reported to be higher in SLE patients compared to healthy individuals, correlating inversely with C3 complement levels (26). Moreover, specific single nucleotide polymorphisms (SNPs) in the TNF-α gene, particularly TNF-376G/A, have been linked to heightened susceptibility to LN, whereas the TNF-308G/A variant appears unrelated to disease risk (27). Despite these findings, the epigenetic regulation bridging these SNPs to altered TNF-α signaling remains poorly understood. While existing studies reinforce TNF-α’s inflammatory contribution in lupus, its mechanistic involvement in Treg dysfunction and vascular endothelial activation warrants in-depth exploration using cell-specific gene deletion approaches (25, 28).

## 3 Th2-associated cytokines in SLE pathogenesis

### 3.1 The IL-4/IL-13 axis in lupus

IL-4 and IL-13 are canonical Th2 cytokines that share significant structural and functional overlap, particularly through co-activation of the STAT6 signaling cascade (29). Both cytokines are secreted by Th2 cells, mast cells, and basophils, and orchestrate humoral immune responses by promoting B cell maturation, IgE class switching, and type 2 inflammation (30, 31). In SLE, IL-4/IL-13 axis displays both immunoregulatory and pathogenic roles (31). In lupus-prone MRL/lpr mice, IL-4 signaling impairs apoptosis of autoreactive B cells. Deocharan et al. (32) demonstrated that IL-4 reduced B220^+^CD19^+^ B cell apoptosis by 53%, correlating with increased anti-dsDNA titers. While serum IL-4 levels are paradoxically lower in SLE patients than in healthy individuals (33), genetic polymorphisms—such as the rs2243250—have been linked to increased SLE susceptibility (34). Similarly, IL-13 contributes to disease progression via the STAT6 pathway, promoting IgE production, renal epithelial CD80 expression, and IL-6 secretion. Clinically, IL-13 levels are higher in active SLE and 3.4-fold higher in LN patients compared to non-LN individuals, correlating with SLEDAI and 24-hour proteinuria (35, 36). Brugos et al. (36) further demonstrated that elevated IL-13 exacerbates immune complex deposition and podocyte dysfunction in LN. Although IL-13 enhances Th2–B cell interactions and drives renal inflammation, its contribution to mesangial proliferation and complement dysregulation remains unclear. Future studies should employ conditional knockout models and spatial transcriptomic approaches to dissect the spatiotemporal contributions of each cytokine in SLE progression.

### 3.2 IL-5–induced B cell dysregulation

IL-5, a prototypical Th2 cytokine released by activated CD4^+^ T cells and mast cells, orchestrates eosinophil recruitment and B cell maturation during immune dysregulation (30). Functional studies have identified its dual immunomodulatory roles. In lupus-prone NZB/W F1 mice, IL-5 overproduction led to increase in proliferating splenic B220^+^IgM^+^ cells and accelerated plasma cell differentiation through BCMA signaling, linking IL-5 to autoreactive B cell expansion in SLE (37). Clinical findings, however, remain inconsistent. Some reports observed markedly elevated IL-5 levels in active SLE patients, correlating with anti-nuclear antibody titers (38, 39), while a larger multicenter cohort identified IL-5 upregulation in only 32% of patients, without a clear association with SLEDAI scores (40). This variability may reflect underlying genetic differences, as IL5RA polymorphisms appear to influence eosinophilic infiltration, though their downstream regulatory axes in SLE are not fully delineated (41). Although IL-5 may contribute to antibody isotype switching (IgA, IgE) and tissue pathology, its dynamic expression patterns and potential synergy with IL-4 and IL-13 warrant further exploration via single-cell transcriptomics and spatial profiling techniques.

### 3.3 IL-6 signaling and clinical heterogeneity

IL-6 is a pivotal mediator of SLE pathogenesis via the JAK–STAT3 axis. Promoter polymorphisms, notably rs1800795 and rs1800796, have been associated with increased SLE susceptibility (42). Clinically, serum IL-6 levels are elevated in SLE and correlate with SLEDAI scores (43). Targeting IL-6 signaling with tocilizumab, an anti-IL-6R monoclonal antibody, significantly reduces anti-dsDNA titers and ameliorates arthritis; however, neutropenia occurred in 30% of patients in early-phase trials (44). Beyond safety concerns, IL-6 inhibition may impair immunological memory: as IL-6 supports B cell maturation, germinal center dynamics, and plasma/memory B cell generation, its blockade can blunt vaccine responses, particularly relevant in SLE populations with elevated infection risk. IL-6 also promotes neutrophil differentiation and survival, and its sustained suppression may weaken host defense against pathogens (45). Diminished vaccine efficacy, notably against influenza and SARS-CoV-2, has been observed in patients on IL-6 inhibitors (46). This therapeutic paradox, attenuation of Th17-driven tissue damage versus disruption of humoral and innate immunity, underscores the need for precision in IL-6–targeted interventions. Future efforts must delineate context-specific signaling to balance efficacy and immunological safety.

### 3.4 IL-10 modulates immune balance in SLE

IL-10 exhibits dual immunomodulatory roles in SLE, primarily via STAT3 signaling downstream of monocytes, macrophages, regulatory B cells, and Th2 cells (47, 48). *In vitro*, IL-10 promotes CD19^+^CD27^+^ memory B cell expansion and elevates anti-dsDNA titers (49), while clinical data show serum IL-10 levels are higher in active SLE and correlate with renal pathology (50). Conversely, IL-10 deficiency aggravates disease: IL-10 knockout mice display a 65% reduction in CD19^+^CD24^hi^CD38^hi^ Bregs and exacerbated proteinuria, underscoring its role in immune tolerance (51). This paradox arises from spatiotemporal and cell-type–specific IL-10Rα expression. In regulatory cells, IL-10 signals through the STAT3–p21 axis to inhibit plasmablast formation, while in autoreactive B cells it enhances BAFF production and survival (52, 53). Early IL-10 secretion may limit inflammation, yet chronic elevation sustains B cell hyperactivity (54, 55). This dynamic modulation is shaped by local cytokine milieus and differential downstream signaling strength. Despite its established relevance to SLE pathogenesis, the spatiotemporal regulation of IL-10, its interplay with type I interferons, and the cell-specific effects require further dissection using targeted gene deletion strategies.

## 4 Th17-associated cytokines in SLE pathogenesis

### 4.1 The IL-17 signaling axis in SLE pathogenesis

IL-17, primarily secreted by Th17 cells, is a central mediator of immunopathology in SLE, bridging innate and adaptive immunity via NF-κB, AP-1, and STAT3 activation (56). Elevated serum IL-17 correlates with SLEDAI scores, particularly in neuropsychiatric SLE, implicating it in neuroimmune dysfunction (57, 58). IL-17 drives pathology through multiple mechanisms: it stimulates keratinocyte-derived IL-6 and IL-8, enhances BAFF expression to sustain autoreactive B cell survival, and promotes NET formation, amplifying DNA antigen exposure and type I IFN production. In MRL/lpr mice, IL-17 knockout reduces germinal center (GC) B cells, anti-dsDNA antibodies, and renal immune complexes (59). Further, IL-17 reinforces Tfh–B cell interactions via the PP2A/CaMK4/ROCK/mTORC1 axis, increasing anti-dsDNA IgG by 3.2-fold, and synergizes with BAFF to activate B cell CD40–NF-κB signaling. By disrupting CXCL12/CXCL13 gradients, it elevates plasma cell retention in GCs by 58%, impairing affinity maturation (60, 61). NET formation, induced 4.7 times more frequently in SLE neutrophils, releases nuclear contents sensed by pDC TLR9, initiating STAT1/IRF7-driven IFN-α feedback loops (62, 63). At the tissue level, IL-17 establishes a reciprocal amplification loop with IL-6: STAT3–RORγt–driven Th17 polarization increases IL-6, which in turn enhances IL-17 secretion from renal tubular epithelial cells, promoting fibrosis via JAK2–STAT3–SOCS3 signaling (64). IL-17A/F, particularly in concert with IL-23, boosts RORγt activity and IL-22 production, driving keratinocyte secretion of CCL20 and MIP-3α (65). Despite its pathogenic significance, the tissue-specific roles of IL-17A versus IL-17F, receptor heterogeneity (IL-17RC/RE), and modulation by gut microbiota-derived metabolites remain poorly defined. IL-17 blockade (brodalumab) shows cutaneous benefit but variable efficacy in LN, likely reflecting microenvironmental variation (66). Future studies employing spatial transcriptomics and single-cell multi-omics are essential to unravel IL-17’s context-specific effects on NETosis, Th17/Treg balance, and metabolic programming within the SLE immune landscape.

### 4.2 Mechanisms of Th17 dysregulation in SLE pathogenesis

Clonal expansion of Th17 cells is central to SLE pathology, influenced by multiple regulatory disruptions (67). Environmental factors, such as gut microbiota imbalance and pathogen-derived signals, promote Th17 polarization by destabilizing T cell homeostasis (56, 68, 69). On the molecular level, persistent STAT3 activation cooperates with TGF-β, driving RORγt-mediated transcriptional reprogramming and boosting the conversion of naïve CD4^+^ T cells to Th17. This also diverts Tregs to Th17-like subsets (70). Pathologically, expanded Th17 cells release IL-17A/F, IL-22, and CCL20, activating endothelial ICAM-1/VEGF pathways, which promotes NET formation and CD138^+^ plasma cell differentiation, leading to a 4.7-fold increase in anti-dsDNA titers (71, 72). In SLE, Th17/Treg ratio was 5.6:1 in peripheral blood, with renal Th17 cells accounting for 35% of CD4^+^ T cells. This contributes to podocyte nephrin downregulation via NFATc1/CaMK4 signaling, worsening proteinuria (73, 74). Despite this understanding, the role of the gut-kidney axis, metabolic reprogramming, and tissue-resident memory Th17 (TRM) dynamics require further investigation using spatial transcriptomics.

### 4.3 Th17/IL-17 axis and its contribution to SLE pathogenesis

The Th17/IL-17 axis plays a central role in SLE immunopathogenesis. Following autoantigen exposure, TLR4 activation by DAMPs induces APC-derived IL-6, IL-23, and TGF-β, promoting STAT3-dependent Th17 differentiation and IL-17A/F production (75, 76). In LN, Th17 cells infiltrate renal tissue via CCL20–CCR6, exhibit enhanced chemotaxis, and drive epithelial–mesenchymal transition through NF-κB and CaMK4, markedly increasing fibrosis (77). Renal biopsies show Th17 enrichment in SLE, correlating with proteinuria (78). IL-17RA/RC expression on plasma cells augments pathogenic IgG and anti-dsDNA autoantibodies via PI3K/Akt/mTOR, with IL-17 stimulation increasing largely (79). Yet, the integration of Th17 activity, Treg dysregulation, and epigenetic remodeling in SLE remains incompletely defined and warrants high-resolution single-cell analyses. IL-23, essential for Th17 polarization, amplifies SLE inflammation via IL-23R/STAT3 signaling. It synergizes with TNF-α to enhance CXCL10 from monocytes and intensify Th1 and Th17 responses through RORγt activation, establishing a self-reinforcing inflammatory loop (80). Elevated IL-23 correlates with SLEDAI scores and is particularly prominent in LN (81). In phase IIb trials, risankizumab (anti–IL-23) achieved nearly 75% lesion reduction in 68% of cutaneous lupus patients, although infection risk increased (82). Moreover, IL-23’s influence on γδT17 trafficking, microbiota-immune crosstalk, and chromatin remodeling remains underexplored. Future investigations integrating single-cell and spatial transcriptomics may uncover novel regulatory axes and therapeutic windows in IL-23–driven SLE pathobiology.

## 5 Emerging cytokine targets in SLE pathogenesis

IL-22, produced by Th17 cells, γδ T cells, and ILC3s, contributes to SLE via STAT3 signaling, promoting SOCS3-mediated MMP-9 induction and podocyte nephrin loss, exacerbating LN progression (83). Serum IL-22 levels are markedly elevated in LN and correlate with proteinuria and fibrosis. In MRL/lpr mice, IL-22 blockade ameliorated renal injury, highlighting therapeutic promise (84). Additionally, microbial dysbiosis and intestinal barrier disruption drive IL-22 overproduction through TLR-IL-23–dependent activation of ILC3s and Th17 cells. Antibiotic-induced microbiota depletion reduces IL-22 and proteinuria, implicating a gut-kidney axis in LN pathogenesis (85). However, the interplay between IL-22, IL-17/IL-23 circuits, and Th17/Treg balance remains incompletely understood. IL-39, a novel IL-12 family heterodimer (p19/Ebi3), is secreted by activated B cells via TLR7–MyD88 pathways and induces BAFF production and plasma cell expansion through STAT1/STAT3 co-activation (86, 87). Murine models show that IL-39 deficiency reduces plasma cell differentiation and renal damage (88). Yet, IL-39 lacks conserved functionality in humans, as recombinant IL-39 fails to activate STAT3 or alter cytokine expression in human PBMCs (89). Its receptor complex (IL-23R/gp130) and biological relevance remain undefined, and no clinical trials have been initiated. Macrophage migration inhibitory factor (MIF), a pleiotropic cytokine, activates the NLRP3 inflammasome via CD74/CXCR4/mTOR signaling, promoting IL-1β release and pyroptosis in renal epithelium (90). Clinically, serum MIF levels correlate with proteinuria and creatinine and inversely with C3, especially in LN (91). IMTO-05, an anti-MIF monoclonal antibody, reduced proteinuria in early trials but was halted due to adverse events and limited enrollment (92). MIF also modulates T cell costimulation, Th17/Treg imbalance, and NET formation, though its mechanistic roles warrant validation through gene-editing studies.

## 6 Therapies targeting IL-17

Targeting IL-17 in SLE involves three primary strategies, including direct neutralization with secukinumab or ixekizumab, achieving 63% renal and 73% skin response but with increased infection risk (61, 77), indirect inhibition via CaMK4 and STAT3 blockade, which suppress IL-17A production and reduce proteinuria through H3K27ac modulation (62, 64, 93), and metabolic reprogramming, which decreases renal IL-17^+^ cells by 61% (94). Combined therapies restore Treg/Th17 balance and lower SLEDAI by 48% (77). However, CaMK4’s widespread expression in non-immune tissues raises off-target toxicity concerns, as current inhibitors lack immune cell specificity. To address this, immune-targeted strategies, such as CD4^+^ T cell–selective liposomes, antibody-drug conjugates, and conditional CRISPR/Cas9 systems, are under development. Novel regulators like miR-125a-3p reduce renal fibrosis by 61% (95), while secukinumab yields 77% reduction in anti-dsDNA titers in refractory LN (96). Challenges persist, including STAT3 inhibitor hepatotoxicity and compensatory IL-23/IL-1β upregulation (97). Future efforts should prioritize multi-omics biomarker integration and tissue-specific delivery systems to enhance precision and minimize systemic toxicity.

## 7 Conclusion

SLE is a multifaceted autoimmune disorder driven by a combination of genetic, environmental, and immune factors. Th1, Th2, and Th17-associated cytokines play central roles in the progression and clinical manifestation of the disease. Despite significant advances in understanding the immunopathology of SLE, including the roles of cytokines like IL-17, IL-23, and IL-10, therapeutic strategies remain limited by issues related to safety and efficacy durability. Targeting cytokines such as IL-17 has shown promise in clinical trials, but challenges such as compensatory upregulation of other inflammatory pathways and potential adverse effects require further exploration. Emerging targets like IL-22 and MIF offer new avenues for intervention, although their mechanisms and interactions within the immune microenvironment need more comprehensive investigation. To achieve precise and effective treatments, future research must focus on integrating multi-omics approaches, identifying reliable biomarkers, and exploring novel delivery systems to tailor therapies to individual patients, minimizing SLE heterogeneity and improving long-term outcomes.
